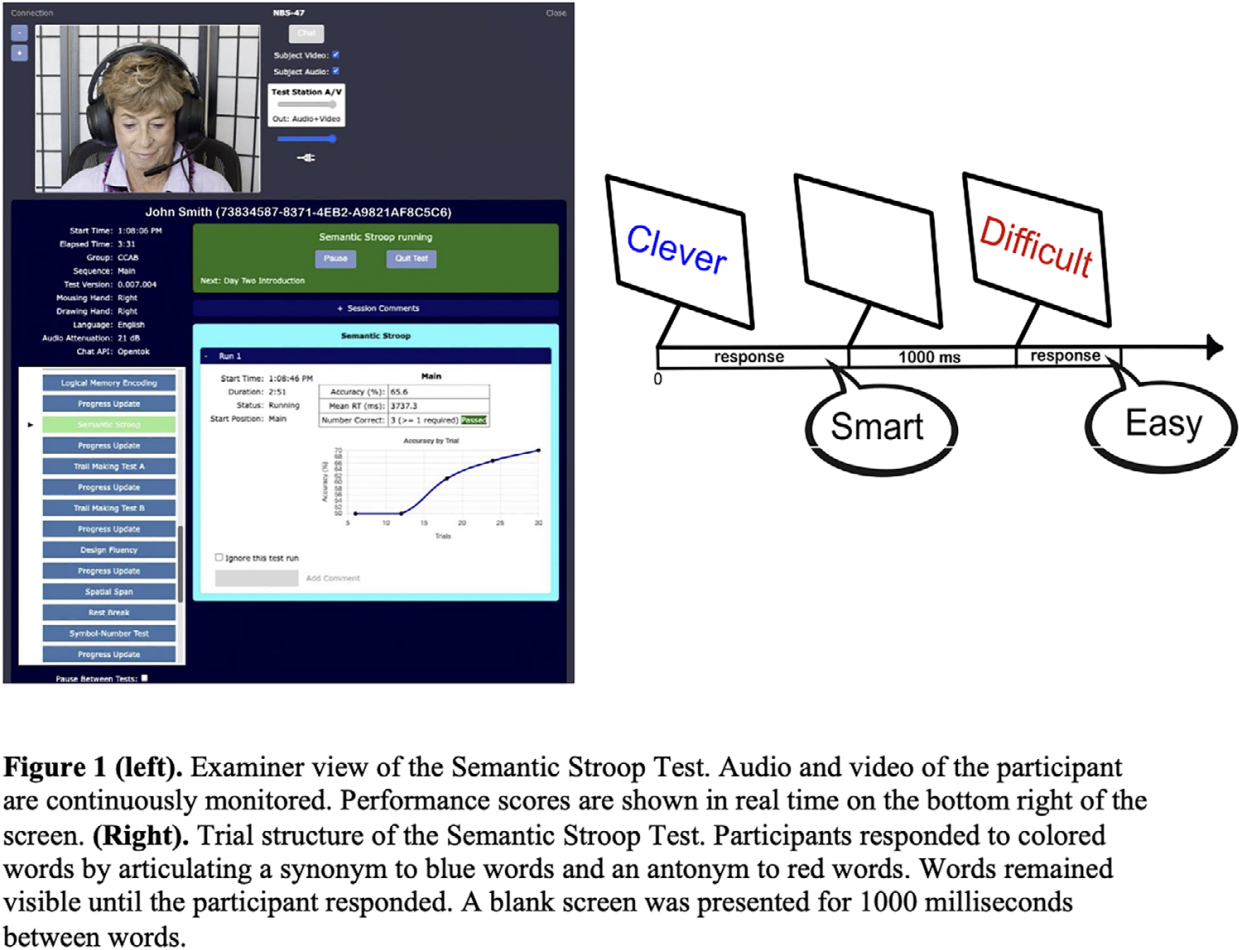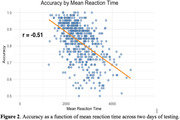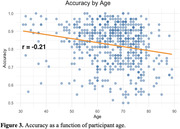# Measuring The Speed and Accuracy of Semantic Retrieval with the Semantic Stroop Task

**DOI:** 10.1002/alz.091683

**Published:** 2025-01-03

**Authors:** Omar Kahly, Kathleen Hall, Juliana Baldo, Krista Schendel, Timothy J Herron, Kristin Geraci, Michael Blank, Isabella Jaramillo, Garrett Williams, Miranda Miranda, Jas M. Chok, Sandy J. Lwi, Brian Curran, Isabella Santavicca, Lexie Thomas, Maria G Spinelli, David K Johnson, David L. Woods

**Affiliations:** ^1^ Neurobehavioral Systems, Inc, Berkeley, CA USA; ^2^ 2093 Addison St, Berkeley, CA USA; ^3^ Veterans Affairs Northern California Health Care System, Martinez, CA USA; ^4^ University of Chicago, Chicago, IL USA; ^5^ UC Davis Alzheimer’s Disease Center, Walnut Creek, CA USA

## Abstract

**Background:**

Semantic memory assessments are sensitive indicators of cognitive decline in pre‐clinical Alzheimer’s Disease (AD), with slowed reaction time and diminished accuracy serving as markers for amyloid accumulation. We introduce the Semantic Stroop Test—a brief (4.3 minute), automated semantic retrieval and executive function task included in the California Cognitive Assessment Battery (CCAB).

**Method:**

The Semantic Stroop Test was administered to 572 healthy adults (50% female, Age = 65.6 years, ±10.6). Participants were tested in their homes using tablet computers. Two sessions were administered on consecutive days, with tests remotely monitored (Figure 1).

Participants were instructed to produce an antonym in response to words in red font and a synonym in response to words in blue font. Sixteen words were presented in random order (Figure 1). Then the same 16 words were presented again in opposite colors. Verbal reaction time (RT) and response accuracy were quantified for each word.

**Result:**

The Semantic Stroop Test showed excellent test‐retest reliability (r = 0.71 accuracy, r = 0.82 RT) and switch failure detection (r = 0.47) in tracking errors. A multiple regression model revealed significant effects of Age and Vocabulary (p<0001) on Accuracy and accounted for 41% of the variance. RT analysis also showed significant effects of both Age and Vocabulary (p<.0001) as well as Education (p<.05), with the model accounting for 30.1% of the variance. Faster RT was associated with higher Accuracy (r = ‐.51) (Figure 2). Furthermore, subjects with consistent sequential responses (e.g., synonym‐synonym) were more accurate than response‐switch trials. Subjects were also more accurate in response to antonym‐based cues than synonyms with 88% vs 79% correct.

**Conclusion:**

The Semantic Stroop Test demonstrates excellent psychometric properties. It reliably captures age‐related cognitive effects through accuracy and speed measures of semantic retrieval while remaining sensitive to executive function processes such as cue‐based switching.